# Ixekizumab improves sleep and work productivity in patients with non-radiographic axial spondyloarthritis: results from the COAST-X trial at 52 weeks

**DOI:** 10.1186/s41927-021-00218-y

**Published:** 2021-09-25

**Authors:** Atul Deodhar, Philip Mease, Helena Marzo-Ortega, Theresa Hunter, David Sandoval, Andris Kronbergs, Steven Lauzon, Ann Leung, Victoria Navarro-Compán

**Affiliations:** 1 Division of Arthritis and Rheumatic Diseases, Oregon Health & Science University, 3181 Sam Jackson Park Rd, Portland, OR 97239 USA; 2 Swedish Medical Center/Providence St. Joseph Health and University of Washington, Seattle, WA USA; 3 NIHR Leeds Biomedical Research Centre, Leeds Teaching Hospitals Trust and LIRMM, University of Leeds, West Yorkshire, Leeds, UK; 4 Eli Lilly and Company, Indianapolis, IN USA; 5 Syneos Health, Morrisville, NC USA; 6 University Hospital La Paz, IdiPaz, Madrid, Spain

**Keywords:** Non-radiographic axial spondyloarthritis, Sleep, Work productivity, Activity impairment, Ixekizumab

## Abstract

**Background:**

Patients with non-radiographic axial spondyloarthritis experience negative impacts on sleep, work productivity, and activity impairment. Ixekizumab, a monoclonal antibody selectively targeting interleukin-17A, has shown efficacy in treating the signs and symptoms of non-radiographic axial spondyloarthritis. This analysis evaluated the effect of ixekizumab treatment on sleep, work productivity, and activity impairment in patients with non-radiographic axial spondyloarthritis.

**Methods:**

COAST-X (NCT02757352) was a 52-week, phase 3, multicenter, randomised placebo-controlled trial evaluating 80-mg ixekizumab every 2 weeks and every 4 weeks in patients with active non-radiographic axial spondyloarthritis. Sleep disturbance was measured with the Jenkins Sleep Evaluation Questionnaire (JSEQ) and analysed using mixed-effects models for repeated measures. Work productivity and activity impairment were measured using the Work Productivity and Activity Impairment Questionnaire for Spondyloarthritis and analysed using analysis of covariance. Absenteeism, presenteeism, and overall work impairment were assessed for patients reporting paid work; activity impairment was assessed regardless of work status.

**Results:**

Overall, patients treated with both dosing regimens of ixekizumab reported numerically greater improvements in sleep than placebo through Week 52. At Weeks 16 and 52, patients treated with ixekizumab every 4 weeks had significantly greater improvements in presenteeism (*p* = 0.007 and *p* = 0.003, respectively) and overall work impairment (*p* = 0.014 and *p* = 0.005, respectively) and numeric improvements in absenteeism than placebo. Patients treated with ixekizumab every 2 weeks had numerically greater improvements in absenteeism, presenteeism, and overall work impairment than placebo. Both dosing regimens of ixekizumab were associated with significantly greater improvements in activity impairment than placebo (ixekizumab every 4 weeks: *p* = 0.003 at Week 16 and *p* = 0.004 at Week 52; ixekizumab every 2 weeks: *p* = 0.007 at Week 16 and *p* = 0.006 at Week 52).

**Conclusions:**

Treatment with ixekizumab improved sleep, work productivity, and activity impairment in patients with nr-axSpA. Improvements in presenteeism and overall work impairment were sustained and consistent in the patients treated with ixekizumab every 4 weeks from Week 16 to Week 52. Improvements in activity impairment were sustained and consistent in both ixekizumab-treated groups from Week 16 to Week 52.

**Trial registration:**

NCT02757352, May 2, 2016.

## Background

Non-radiographic axial spondyloarthritis (nr-axSpA), part of the axial spondyloarthritis (axSpA) disease spectrum, is classified in patients without definitive changes in the sacroiliac joints as established by the modified New York (mNY) criteria [1–3]. Patients with nr-axSpA lack definite sacroiliitis on plain radiographs but could show inflammatory changes of the sacroiliac joints on magnetic resonance imaging (MRI) [4]. Alternatively, patients with nr-axSpA can also be classified based on HLA B-27 positivity and two additional spondyloarthritis features [4]. Nr-axSpA and radiographic axSpA (r-axSpA), traditionally known as ankylosing spondylitis (AS), are different axSpA disease states sharing similar, significant disease burdens as measured by patient-reported outcomes (PROs) [1–3, 5].

AxSpA negatively affects patients’ ability to sleep and work. Sleep and fatigue are considered important aspects of health for patients with axSpA, and poor sleep contributes to fatigue [6–8]. Fatigue, in turn, is associated with work productivity and activity impairment [9]. Patients with axSpA who report employment are likely to encounter increased absences away from work, decreased productivity while at work, and/or early retirement [10–12]. In addition, loss of employment is associated with worse PROs [12]. Patients’ reduced ability to work from the burden of axSpA can result in significant impacts on society and the economy as axSpA is usually diagnosed at a relatively young age [10].

Many patients with axSpA who receive treatment still experience disease-related burdens in different aspects of health-related quality of life (HRQoL), which include sleep, work productivity, and activity impairment [10]. While tumor necrosis factor inhibitors (TNFi) have been found to improve axSpA patients’ HRQoL, not all patients receiving TNFi tolerate or respond to treatment, suggesting that treatments with different mechanisms of action may be indicated in some patients [4, 10, 13]. Ixekizumab is a high-affinity monoclonal antibody that selectively targets interleukin-17A. COAST-X was the pivotal randomised phase 3 trial that proved the efficacy of ixekizumab for treating nr-axSpA with objective signs of inflammation [1]. The present analysis used data from COAST-X to assess the effect of ixekizumab treatment for 52 weeks on sleep, work productivity, and activity impairment in patients with active nr-axSpA.

## Methods

### Study design

Details on the design of the COAST-X trial (NCT02757352) have been published previously [1]. COAST-X was a phase 3, randomised, placebo-controlled trial evaluating 80-mg ixekizumab every 2 weeks (Q2W) and every 4 weeks (Q4W) for 52 weeks.

### Patients

Patients enrolled in COAST-X were adults with a physician’s diagnosis of nr-axSpA. Enrolled patients fulfilled the Assessment of SpondyloArthritis International Society (ASAS) criteria for axSpA with active disease despite at least 12 weeks of previous therapy with NSAIDs with an inadequate response to at least 2 NSAIDs or a history of intolerance to NSAIDs. Patients who had confirmed sacroiliitis on centrally read x-rays (following mNY criteria) were excluded [1]. Enrollment required active disease at screening and baseline as well as objective signs of inflammation. Active disease was defined as a Bath Ankylosing Spondylitis Disease Index (BASDAI) score of at least 4 and a total back pain score of at least 4. Objective signs of inflammation were defined as evidence of elevated C-reactive protein (CRP) greater than 5 mg/L and/or the presence of sacroiliitis (following the ASAS definition) on centrally read MRI [1]. Patients could continue stable background medications, including NSAIDs, conventional synthetic disease-modifying antirheumatic drugs (csDMARDs), corticosteroids, and analgesics, during the trial [1]. Before participating in the study, enrolled patients gave written informed consent. The COAST-X trial was conducted in accordance with the standards of the Declaration of Helsinki and Good Clinical Practice Guidelines (CPMP/ICH/135/95) [1].

### Randomisation and blinding

COAST-X enrolled 303 patients. Patients were randomised 1:1:1 to receive placebo Q2W (*N* = 105), 80-mg ixekizumab Q4W (*N* = 96), or 80-mg ixekizumab Q2W (*N* = 102) by subcutaneous injection [1]. Randomisation was stratified by country and screening MRI/CRP status for group comparability. There were 3 MRI/CRP statuses: positive MRI and elevated CRP, positive MRI and non-elevated CRP, and negative MRI and elevated CRP. The randomised, blinded treatment period lasted from Weeks 0 to 52. Background medications could be adjusted from Weeks 16 to 44. Patients could also switch to open-label ixekizumab Q2W or subsequent TNFi treatment following the clinical judgment of investigators from Weeks 16 to 44. No specific switch criteria were predefined. If a patient switched to open-label treatment, the patient continued to be followed for the trial’s duration, and both the patient and investigator continued to be blinded to the patient’s original treatment group.

### Outcome measures

Sleep problems were measured by the Jenkins Sleep Evaluation Questionnaire (JSEQ). JSEQ is comprised of 4 items that are related to trouble falling asleep, waking up numerous times throughout the night, trouble remaining asleep and/or waking up too early, and waking up after a usual amount of sleep but still being affected by fatigue [8]. Patients report how many days they experience each of the above problems over the past month. Scoring uses a 6-point Likert Scale that ranges from 0 (“no days”) to 5 (“22–30 days”). Total JSEQ scores range from 0 to 20. Higher scores indicate more sleep disturbance [8]. During the COAST-X trial, patients’ JSEQ scores were measured at baseline (Week 0) and Weeks 8, 16, 36, and 52.

Work productivity and activity impairment was measured with the Work Productivity and Activity Impairment Questionnaire for Spondyloarthritis (WPAI-SpA). WPAI-SpA is validated in in patients with AS [14]. WPAI-SpA is comprised of 6 questions evaluating patients’ experiences during the previous week. Employment status, missed hours from work due to causes related to spondyloarthritis (SpA), missed hours due to causes not related to SpA, hours actually worked, how SpA impacted work productivity while at work, and how SpA impacted activities while not at work. WPAI-SpA scores are recorded in 4 domains, which are: percentage of absenteeism, percentage of presenteeism (reduced productivity while at work), overall work impairment (combining absenteeism and presenteeism), and activity impairment (percentage of impairment in activities away from work). Absenteeism, presenteeism, and overall work impairment are recorded for patients who report part- or full-time employment (paid work). Activity impairment is recorded for all patients regardless of their employment status. Higher WPAI-SpA scores correspond to worse work productivity and activity impairment. During the COAST-X trial, WPAI-SpA scores were measured at baseline (Week 0) and at Weeks 16 and 52.

### Statistical analyses

Data were analysed from the intent-to-treat population. Ixekizumab Q4W and ixekizumab Q2W (both 80 mg) were compared to placebo for the duration of the blinded treatment period from Weeks 0 to 52 and before any biologic switches to ixekizumab Q2W or TNFi. Only data up to the time of switch were included in these analyses. Changes from baseline were analysed as least squares means (LSMs) with standard errors (SEs). Comparisons between changes from baseline for JSEQ scores in different treatment groups were conducted using mixed-effects models for repeated measures (MMRM). The MMRM model used to analyse JSEQ scores included treatment, geographic region, screening MRI/CRP status, baseline value, visit, baseline value-by-visit, and treatment-by-visit interactions as fixed factors. No imputation for missing data was performed for JSEQ analyses. Comparisons between changes from baseline for WPAI-SpA scores in different treatment groups were conducted using analysis of covariance (ANCOVA). The ANCOVA model used to analyse WPAI-SpA scores included treatment, geographic region, screening MRI/CRP status, and baseline value as factors. Modified baseline observation carried forward (mBOCF) was used to impute missing data when analysing WPAI-SpA scores. All analyses were conducted with SAS 9.4. SAS was used to generate residual plots to confirm normality of residuals.

## Results

### Baseline characteristics

A total of 303 patients were enrolled in the COAST-X trial. Baseline characteristics were similar among the different treatment groups and are detailed in Table 1.

**Table 1:** Baseline characteristics of patients enrolled in COAST-X

	PBO (N = 105)	IXE Q4W (*N* = 96)	IXE Q2W (*N* = 102)	Total (*N* = 303)
Age, years	39.9 (12.4)	40.9 (14.5)	40.0 (12.0)	40.3 (12.9)
Male, n (%)	44 (41.9)	50 (52.1)	49 (48.0)	143 (47.2)
Weight, kg	75.8 (18.4)	79.5 (16.5)	77.3 (16.6)	77.5 (17.2)
Duration of nr-axSpA symptoms, years	10.1 (8.3)	11.3 (10.7)	10.6 (10.1)	10.7 (9.7)
Time since nr-axSpA diagnosis, years	3.1 (4.5)	4.2 (5.5)	3.4 (4.6)	3.6 (4.9)
CRP, mg/L	14.3 (24.4)	12.4 (18.0)	12.1 (17.8)	12.9 (20.4)
Screening MRI/CRP status, n (%)				
Positive MRI and elevated CRP	38 (36.2)	30 (31.3)	39 (38.2)	107 (35.3)
Positive MRI and nonelevated CRP	40 (38.1)	36 (37.5)	34 (33.3)	110 (36.3)
Negative MRI and elevated CRP	26 (24.8)	30 (31.3)	28 (27.5)	84 (27.7)
Therapy, n (%)				
Current csDMARD use, including MTX	36 (34.3)	40 (41.7)	42 (41.2)	118 (38.9)
Current MTX use	17 (16.2)	17 (17.7)	15 (14.7)	49 (16.2)
Current NSAID/COX-2 inhibitor use	96 (91.4)	81 (84.4)	95 (93.1)	272 (89.8)
ASDAS-CRP	3.8 (0.9)	3.8 (0.8)	3.9 (0.8)	3.8 (0.8)
BASDAI	7.2 (1.5)	7.0 (1.5)	7.3 (1.3)	7.2 (1.4)
Sleep (JSEQ)	9.4 (5.6)	9.4 (5.3)	10.4 (5.3)	9.7 (5.4)
Reported part- or full-time work, n (%)	69 (65.7)	61 (63.5)	66 (64.7)	196 (64.7)
Work productivity (WPAI-SpA)				
Absenteeism	15.6 (26.8)^a^	12.1 (27.0)^b^	16.1 (26.5)^c^	14.7 (26.7)^d^
Presenteeism*	62.1 (18.7)^e^	50.2 (22.3)^f^	56.3 (24.8)^g^	56.7 (22.4)^h^
Overall work impairment*	65.6 (20.2)^e^	51.9 (22.8)^f^	59.3 (26.4)^g^	59.5 (23.7)^h^
Activity impairment	66.4 (20.4)	63.3 (21.5)	66.1 (20.7)	65.3 (20.8)

^a^N = 65; ^b^N = 53; ^c^N = 60; ^d^N = 178; ^e^N = 62; ^f^N = 49; ^g^N = 57; ^h^N = 168

Data are mean (SD) unless otherwise stated

*P* values from Fisher’s exact test for categorical data (Monte Carlo estimate of exact *p*-value was used for categorical values that have more than 2 categories) and analysis of variance with treatment as a factor for continuous data. *p < 0.05

*ASDAS-CRP* Ankylosing Spondylitis Disease Activity Score with C-Reactive Protein; *BASDAI* Bath Ankylosing Spondylitis Disease Activity Index; *csDMARD* Conventional synthetic disease-modifying antirheumatic drug; *COX-2* Cyclooxygenase-2; *CRP* C-reactive protein; *IXE Q2W* 80-mg ixekizumab every 2 weeks; *IXE Q4W* 80-mg ixekizumab every 4 weeks; *JSEQ* Jenkins Sleep Evaluation Questionnaire; *MRI* Magnetic resonance imaging; *MTX* Methotrexate; *n* number of patients in a subcategory; *N* Number of patients in the analysis category; *NRS* Numeric rating score; *nr-axSpA* nonradiographic axial spondyloarthritis; *PBO* Placebo; *PtGA* Patient’s Global Assessment of Disease Activity; *SD* Standard deviation; *WPAI-SpA* Work Productivity and Activity Impairment Questionnaire for Ankylosing Spondylitis

Patients’ mean age was 40.3 years (SD 12.9). The mean duration of nr-axSpA symptoms was 10.7 years (SD 9.7). At baseline (Week 0), 196 patients (64.7%) reported part- or full-time employment.

### Changes from baseline in sleep

**Fig. 1:**
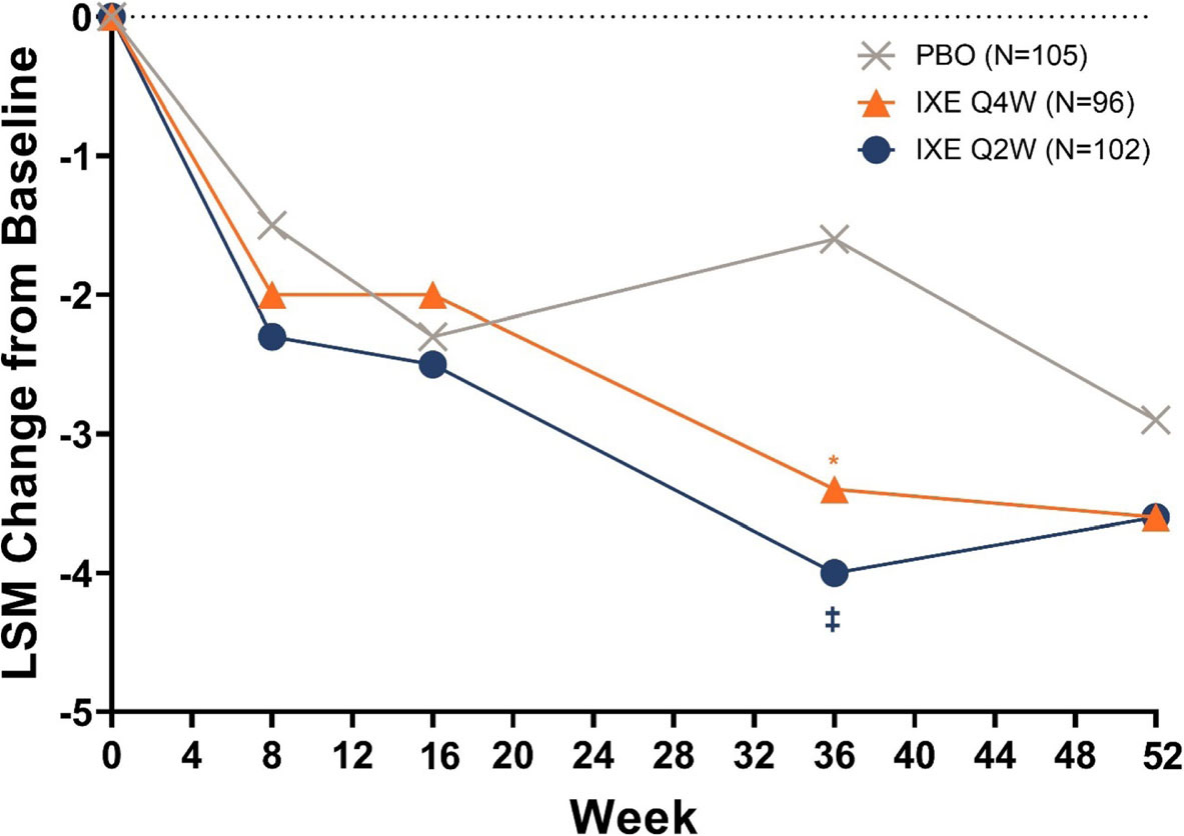
Changes from baseline in sleep as measured by the Jenkins Sleep Evaluation Questionnaire Values are LSM from MMRM. Week 8: PBO, Nx = 101; IXE Q4W, Nx = 95; IXE Q2W, Nx = 101. Week 16: PBO, Nx = 99; IXE Q4W, Nx = 96; IXE Q2W, Nx = 98. Week 36: PBO, Nx = 39; IXE Q4W, Nx = 56; IXE Q2W, Nx = 58. Week 52: PBO, Nx = 34; IXE Q4W, Nx = 53; IXE Q2W, Nx = 52. *P* values were from MMRM (treatment vs. placebo). * = *p* < 0.05, ‡ = *p* < 0.01, † = *p* < 0.001. IXE Q2W = 80-mg ixekizumab every 2 weeks; IXE Q4W = 80-mg ixekizumab every 4 weeks; IXE Q4W = 80-mg ixekizumab every 2 weeks; LSM = least squares mean; MMRM = mixed-effect model repeated measure; N = number of patients in the treatment group; Nx = number of patients with non-missing values; PBO = placebo

Overall, patients treated with ixekizumab Q4W and Q2W had numerically greater improvements in total JSEQ scores compared to placebo (Fig. 1).

At Week 36, patients treated with ixekizumab Q4W and Q2W had significantly greater improvements in JSEQ scores compared to placebo (*p* = 0.015 and *p* = 0.002 for ixekizumab Q4W and Q2W, respectively, versus placebo) (Fig. 1). Numeric improvements in JSEQ in the ixekizumab treatment groups were sustained and consistent to Week 52 (Fig. 1).

### Changes from baseline in absenteeism, presenteeism, and overall work impairment

**Fig. 2:**
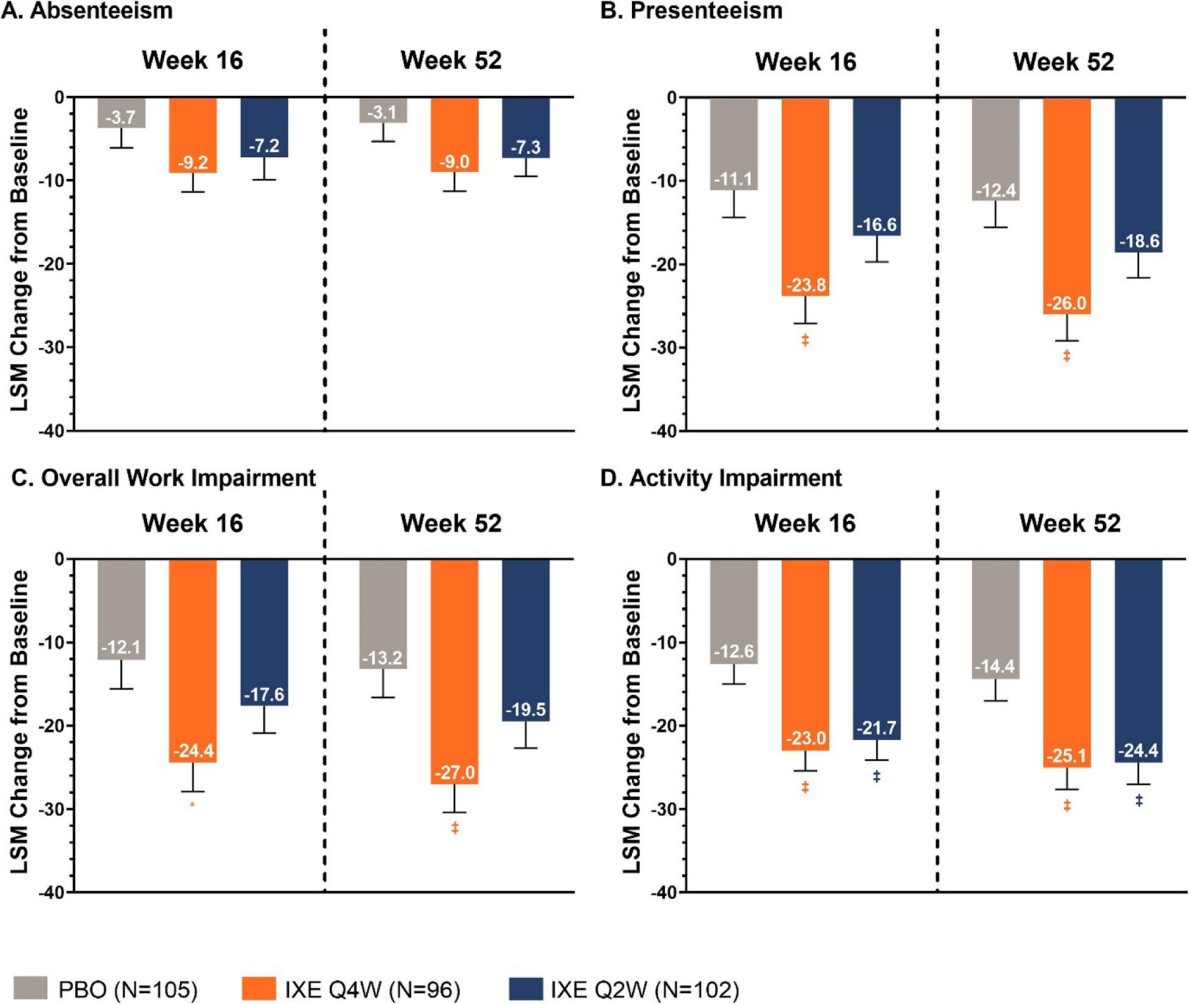
Changes from baseline in Work Productivity and Activity Index Spondyloarthritis A) Absenteeism, B) Presenteeism, C) Overall Work Impairment, and D) Activity Impairment at Weeks 16 and 52 Values are LSM (SE) from mBOCF ANCOVA. Absenteeism, presenteeism, and overall work impairment were measured in patients reporting part- or full-time work. Absenteeism at Week 16: PBO, Nx = 50; IXE Q4W, Nx = 50; IXE Q2W, Nx = 53. Absenteeism at Week 52: PBO, Nx = 52; IXE Q4W, Nx = 50; IXE Q2W, Nx = 55. Presenteeism and Overall Work Impairment at Week 16: PBO, Nx = 47; IXE Q4W, Nx = 46; IXE Q2W = 50. Presenteeism and Overall Work Impairment at Week 52: PBO, Nx = 49; IXE Q4W, Nx = 46; IXE Q2W, Nx = 52. Activity Impairment at Weeks 16 and 52: PBO, Nx = 101; IXE Q4W, Nx = 96; IXE Q2W, Nx = 101. P values were from ANCOVA (treatment vs. placebo). * = p < 0.05, ‡ = p < 0.01, † = p < 0.001. ANCOVA = analysis of covariance; IXE Q2W = 80-mg ixekizumab every 2 weeks; IXE Q4W = 80-mg ixekizumab every 4 weeks; IXE Q4W = 80-mg ixekizumab every 2 weeks; LSM = least squares mean; mBOCF = modified baseline observation carried forward; N = number of patients in the treatment group; Nx = number of patients with non-missing values; PBO = placebo

Patients reporting part- or full-time employment were assessed for WPAI-SpA absenteeism, presenteeism, and overall work impairment. Patients treated with both ixekizumab dosing regimens experienced numerically greater improvements from baseline in absenteeism, presenteeism, and overall work impairment at Weeks 16 and 52 compared to placebo (Fig. 2).

The differences in changes from baseline in presenteeism and overall work impairment were significant for patients treated with ixekizumab Q4W versus placebo at Weeks 16 and 52. For presenteeism, the LSM difference between ixekizumab Q4W and placebo was − 12.7 (SE 4.6; *p* = 0.007) at Week 16, and the LSM difference was − 13.6 (SE 4.6; *p* = 0.003) at Week 52. For overall work impairment, the LSM difference between ixekizumab Q4W and placebo was − 12.3 (SE 4.9) at Week 16 (*p* = 0.014), and the LSM difference was − 13.8 (SE 4.8) at Week 52 (*p* = 0.005).

### Changes from baseline in activity impairment

Activity impairment was assessed regardless of employment status. Patients treated with both ixekizumab dosing regimens experienced significantly greater improvements in activity impairment at Weeks 16 and 52 compared to placebo (Fig. 2). At Week 16, the LSM difference between ixekizumab Q4W and placebo was − 10.4 (SE 3.4; *p* = 0.003), and the LSM difference between ixekizumab Q2W and placebo was − 9.1 (SE 3.4; *p* = 0.007). At Week 52, the LSM difference between ixekizumab Q4W and placebo was − 10.6 (SE 3.7; *p* = 0.004), and the LSM difference between ixekizumab Q2W and placebo was − 10.0 (SE 3.6; *p* = 0.006).

## Discussion

In this study, patients with nr-axSpA treated with ixekizumab reported improved sleep, work productivity, and activity impairment. Primary results of the COAST-X trial have established the efficacy of ixekizumab in treating the signs of symptoms of nr-axSpA as demonstrated by significant improvements in ASAS40 response as early as Week 1 [1].

Patients with nr-axSpA are more likely to report poor sleep than the general population, and patients with nr-axSpA have reported worse sleep disturbance at baseline compared to patients with r-axSpA [15–17]. The baseline mean JSEQ score for patients naïve to biologic disease-modifying antirheumatic drugs (bDMARDs) with nr-axSpA enrolled in the COAST-X trial was slightly higher than the scores for bDMARD-naïve patients with r-axSpA enrolled in the COAST-V trial, which may suggest worse sleep disturbance in bDMARD-naïve patients with nr-axSpA [1, 18]. However, TNFi-experienced patients with r-axSpA enrolled in COAST-W reported higher baseline JSEQ scores than bDMARD-naïve patients enrolled in COAST-X and COAST-V [18]. Factors contributing to the observation of greater sleep disturbance in nr-axSpA compared to r-axSpA may include a greater proportion of women being affected by nr-axSpA as well as a greater prevalence of diagnosed fibromyalgia in nr-axSpA patients [19]. Central sensitization, which is more common in female patients with axSpA and in patients with fibromyalgia, can also contribute to sleep disturbance [20]. Sleep disruption due to disease activity has also been tied to work impairment [15, 21]. Because sleep disruptions may exacerbate chronic inflammatory conditions or serve a marker of underlying inflammation, sleep quality could serve as a proxy for disease activity [15].

While there is not yet a full understanding of nr-axSpA’s impact on work disability and economic consequences, nr-axSpA’s effects are estimated to be equivalent to those of r-axSpA because both disease states share similar severity in disease activity, pain, and fatigue [11, 16]. Baseline absenteeism, overall work impairment, and activity impairment scores were slightly higher in bDMARD-naïve patients with nr-axSpA enrolled in the COAST-X trial compared to the bDMARD-naïve patients with r-axSpA enrolled in the COAST-V trial [22]. TNFi-experienced patients with r-axSpA enrolled in COAST-W reported higher baseline WPAI-SpA scores across all domains than bDMARD-naïve patients enrolled in COAST-X and COAST-V [22]. As described above, central sensitization can affect work impairment because it can worsen sleep disruption [19, 20].

Maintaining employment is an important factor in patients’ quality of life, influencing additional psychosocial, societal, and financial impacts [14, 23, 24]. In a 2015 study evaluating the baseline burden of early nr-axSpA and the effect of treatment with etanercept, 69.7% (129/185) of patients were employed at baseline; a similar proportion was found in the present analysis with 64.7% (196/303) of patients enrolled in COAST-X reporting part- or full-time employment [16]. European patients with nr-axSpA had greater work productivity loss compared with the general population, and bDMARD-naïve patients reported worse presenteeism, overall work impairment, and activity impairment than bDMARD-experienced patients [25].

Fatigue and sleep are inherently linked, and patients with axSpA consider both important for overall health [6]. Results from a study of patients with AS and rheumatoid arthritis (RA) treated with etanercept showed a significant association between fatigue and presenteeism and activity impairment (but not overall work impairment or absenteeism) in patients with AS as well as an association between fatigue and all WPAI-SpA domains in patients with RA [9]. A study of patients with axSpA in the United Kingdom found an association between absenteeism and worse fatigue, which was more likely in those with nr-axSpA; presenteeism, work impairment, and activity impairment were also associated with higher levels of fatigue [26]. In another study using data from the British Society for Rheumatology Biologics Register, fatigue was associated with absenteeism and presenteeism in patients with axSpA [21]. Results from the European Map of Axial Spondyloarthritis survey suggested that worse patient-reported outcomes, particularly greater disease activity and psychological distress, were associated with work-related issues [27].

In a recent study using data from 2 trials of patients with r-axSpA naïve to bDMARDs and those who were intolerant of or who had inadequate response to TNFi (COAST-V and COAST-W, respectively), treatment with ixekizumab improved work productivity and activity impairment as measured by WPAI-SpA with sustained and consistent results through Week 52 [22]. In the context of nr-axSpA, the efficacy of ixekizumab treatment in improving signs and symptoms in the COAST-X trial has been demonstrated [1]. A recent study using COAST-X trial data demonstrated that treatment with ixekizumab improved patient-reported outcomes in nr-axSpA, including patient global disease activity, spinal pain, function, stiffness, fatigue, and spinal pain at night [13]. An additional recent study using COAST-X trial data showed that treatment with ixekizumab improved patient-reported functioning and health in nr-axSpA [28]. Overall, these data suggest that ixekizumab has significant efficacy on patient-reported outcomes across the axSpA disease spectrum. Beyond axSpA, ixekizumab has also shown effectiveness in improving work productivity in patients with moderate-to-severe plaque psoriasis and psoriatic arthritis [29–31].

The results of this study show improvements in sleep, work productivity, and activity impairment and are consistent with previously reported results demonstrating the efficacy of ixekizumab in treating nr-axSpA [1, 13]. Collectively, these results suggest treatment with ixekizumab can improve patient-reported outcomes as well as overall HRQoL in the specific context of nr-axSpA.

Limitations of this study include the absence of specific predefined criteria to switch to open label, which followed regulatory requirements. At investigator discretion, substantial proportions of patients who achieved clinical response were switched from their blinded ixekizumab treatment to open-label ixekizumab and were considered non-responders. In addition, while over 60% of patients enrolled in COAST-X reported paid work, WPAI-SpA requires part- or full-time employment for absenteeism, presenteeism, and overall work impairment scores. The limited number of patients reporting paid work may have impacted analyses determining the statistical significance of changes from baseline in WPAI-SpA scores, particularly in absenteeism.

Strengths of the COAST-X trial included the enrollment of patients from multiple global regions. In addition, enrolled patients had objective signs of inflammation confirmed by screening MRI/CRP status. Patients enrolled in COAST-X also experienced disease symptoms that lasted over 10 years without transition to r-axSpA. This analysis used scores from the validated instruments JSEQ and WPAI-SpA. The presence and consistency of a placebo arm through Week 52 allowed for a true comparison of the effects of ixekizumab against placebo on sleep, work productivity, and activity impairment. COAST-X met ethical requirements by allowing patients to modify background therapy or switch to open-label ixekizumab Q2W after Week 16 (at investigator discretion).

## Conclusions

Patients with active nr-axSpA treated with ixekizumab reported improvements in sleep disturbance, work productivity, and activity impairment compared to those receiving placebo. Numeric improvements in sleep were observed from Weeks 8 to 52. Significant improvements in presenteeism and overall work impairment were sustained and consistent in patients treated with ixekizumab Q4W from Week 16 to Week 52, and significant improvements in activity impairment were sustained and consistent in patients treated with ixekizumab Q4W and Q2W from Week 16 to Week 52.

## Data Availability

Data on all individual participant data collected during the trial (except pharmacokinetic or genetic data) are available upon request. Access is granted after approval by an independent review committee and after receiving a signed data sharing agreement. Please submit any requests for data online.

## Abbreviations

**ANCOVA**: analysis of covariance
**AS**: ankylosing spondylitis
**ASAS**: Assessment of SpondyloArthritis International Society
**axSpA**: axial spondyloarthritis
**BASDAI**: Bath Ankylosing Spondylitis Disease Activity Index
**bDMARD**: biologic disease-modifying antirheumatic drug
**CRP**: C-reactive protein
**csDMARD**: conventional synthetic disease-modifying antirheumatic drug
**HRQoL**: health-related quality of life
**JSEQ**: Jenkins Sleep Evaluation Questionnaire
**LSM**: least squares mean
**MMRM**: mixed-effects model for repeated measures
**mNY**: modified New York
**MRI**: magnetic resonance imaging
**nr-axSpA**: non-radiographic axial spondyloarthritis
**NSAID**: non-steroidal anti-inflammatory drug
**PRO**: patient-reported outcome
**Q2W**: every 2 weeks
**Q4W**: every 4 weeks
**RA**: rheumatoid arthritis
**r-axSpA**: radiographic axial spondyloarthritis
**SE**: standard error
**TNFi**: tumor necrosis factor inhibitors
**WPAI-SpA**: Work Productivity and Activity Impairment Questionnaire for Spondyloarthritis
